# Effects of Acid and Salt Solutions on the Pasting, Rheology and Texture of Lotus Root Starch–Konjac Glucomannan Mixture

**DOI:** 10.3390/polym9120695

**Published:** 2017-12-10

**Authors:** Fusheng Zhang, Min Liu, Fang Mo, Meixia Zhang, Jiong Zheng

**Affiliations:** 1 College of Food Science, Southwest University, Chongqing 400715, China; zfs830804@163.com (F.Z.); LiuMin1912@163.com (M.L.); mofang921212@163.com (F.M.); 2 School of Forestry and Life Science, Chongqing University of Arts and Sciences, Chongqing 402160, China; fenghuodianzmx@163.com

**Keywords:** lotus root starch–konjac glucomannan mixture, acid, saline, properties, microstructure

## Abstract

To explore the functional properties of mixed biopolymer systems affected by acid and salts. The effects of acid and salt solutions (i.e., NaCl, KCl and CaCl_2_) on the pasting, rheology, texture and microstructure of lotus root starch–konjac glucomannan (LRS/KGM) mixtures were assessed. Acid (citric acid buffer) treatment worsened the pasting (except for breakdown viscosity), rheological (except for fluid index), and textural properties, thereby inhibiting retrogradation, weakening pseudoplasticity and thickening, and reducing mixture viscoelasticity. Furthermore, it led to destructive ruptures and large pores in the internal microstructure. Salt treatment worsened the pasting properties (except for setback viscosity), thus inhibiting retrogradation and weakening pseudoplasticity, but enhanced the rheological properties, improving thickening and fluctuating viscoelasticity of the mixture. Moreover, salt addition decreased the hardness while increasing mixture cohesiveness, and modified the elasticity, adhesiveness and internal microstructure in a salt type- and concentration-dependent manner. A salt solution concentration of 0.5 mol/L NaCl, 0.1 mol/L KCl, and 0.5 mol/L CaCl_2_ led to the mixture with the best texture and gel network.

## 1. Introduction

Lotus root is an important and edible aquatic vegetable widely distributed in China, Japan and Southeast Asia. Lotus roots have crisp tissues and a bright color, are rich in nutrients (e.g., starch, protein, cellulose, polyphenolic substances, and antioxidants), and have considerable medicinal properties [1]. Given their high brittleness, lotus root starch (LRS) particles are easily crushed or deformed during heating and agitating but do not readily form a gel after cooling [2]. During food processing, LRS is often combined with hydrophilic colloids to overcome the limitations of native starch processing [3], thereby increasing the quality and stability of LRS-based food products.

Konjac glucomannan (KGM) is a hydrophilic colloid with high tightness and a branched structure [4,5], with many excellent functional characteristics and properties, including hydrophilicity, thickening, gelation, film formation, and bacterial resistance [6,7], as well as being highly biodegradable. Given the above properties, KGM and some similar hydrophilic colloids have been extensively studied in the manufacturing of gel food, beverages, packaging films, and drug carriers, etc. [8,9,10,11].

Generally, when starch and hydrophilic colloids are combined in the appropriate proportions, they exert a synergistic effect on the rheology, pasting, texture and other properties of foods [12,13,14]. Moreover, in many food production processes, starch/hydrophilic colloid mixtures are often used in acid and salinity environments that significantly affect the mixture properties. Significant effects of salt on the gelatinization and viscosity of a corn starch–guar gum mixture and sweet potato starch–xanthan gum mixture, according to the salt type and concentration, have been observed [15,16]. Various studies have also highlighted that salts exert a great influence on the gelatinization, thermodynamics, and rheological properties of a rice starch/hydrophilic colloid mixture [17,18]. Further, the addition of salt enhanced the viscosity of the cassava starch–xanthan gum mixture, whereas acid worsened the viscosity and freeze–thaw stability of the mixture [19,20].

Nevertheless, acid and salinity are known to exert a significant effect on starch/hydrophilic colloid mixture properties, there are no systematic papers to date assessing their effects on pasting, rheology, and texture of LRS/KGM mixture foods. Therefore, the aim of this study was to investigate the effect of acid and salinity on the properties of the LRS/KGM mixture, and provide some reference for the application of starch/hydrophilic colloid mixtures in acid or saline foods.

## 2. Materials and Methods

### 2.1. Materials

LRS (water content = 12%, amylose content = 29% and amylopectin content = 56%) was purchased from Hubei Aihe Food Co., Ltd., Wuhan, China. KGM (water content = 8%) was obtained from Zibo Zhongxuan Biochemical Co., Ltd., Shandong, China. NaCl, KCl, CaCl_2_, citric acid, and sodium dihydrogen phosphate (all of analytical purity) were purchased from ChengDu KeLong Chemical Reagent Plant, Chengdu, China.

### 2.2. Pasting Test

Trial test results (data not shown) showed that the pasting, rheology and texture properties of the LRS/KGM mixture were best at a LRS/KGM ratio of 8.5:1.5 (*M/M*) using the following measured methods. Therefore, LRS/KGM mixtures at this ratio were used for all subsequent experiments. With reference to the method of Gil and Yoo [15], with slight modifications, the analysis of pasting properties was performed using the Rapid Visco Analyzer (RVA-TecMaster, Perten Instruments, New South Wales, Australia) with a RVA aluminum box. Firstly, the solutions were adjusted to pH 3 and 7 using citric acid-containing phosphate buffer, then weighed LRS/KGM was then mixed with the solutions in an aluminum box, forming a 6 g/100 g (mass fraction) suspension, to determine the effect of pH on the LRS/KGM system. The weighed LRS/KGM was mixed with 0, 0.1, 0.5 and 1 mol/L NaCl, KCl, and CaCl_2_ solutions in aluminum box, forming a 6 g/100 g (mass fraction) suspension, to test the effects of salinity on the LRS/KGM system. The standard test program 2 was used, maintaining the temperature at 50 °C for 1 min, decreasing the rotation speed from 960 r/min to a stable 160 r/min, increasing the temperature from 50 °C to 95 °C over 7.5 min and maintaining it for 5 min, and finally decreasing the temperature to 50 °C over 7.5 min and maintaining it for 2 min. The instrument automatically drew the pasting curve.

### 2.3. Rheology Test

With reference to the improved method of Hong et al. [19], RVA-gelatinized samples were placed on the rheometer (DHR-1, TA Instruments, New Castle, DE, USA). The plate–plate measurement was used to test the rheology using a plate diameter of 40 mm, and the interval was set to 1 mm. Samples were changed for each test and balanced for 5 min to remove residual stress on the loading process and ensure a constant temperature.

Static rheological properties: The temperature was maintained at 25 °C and the shear rate was initially increased from 0 s^−1^ to 300 s^−1^ and then decreased from 300 s^−1^ to 0 s^−1^. Herein, regression fitting of static shear data points was conducted based on the power law, as follows:

$$\tau =K\gamma^{n}$$

where *τ* is the shear stress (Pa), *γ* is the shear rate (s^−1^), *n* is the fluid index, and *K* is the consistency coefficient (Pa·s^n^).

Dynamic viscoelasticity test: The linear viscoelastic region was tested at 25 °C. The changes in stored energy modulus (*G*′), loss modulus (*G*″), and loss tangent (tan δ), when the scanning stress was fixed at 1% and at an oscillation frequency range of 0.1–10 Hz, were determined.

Dynamic time scanning: The changes in storage modulus (*G*′) and tan δ of the samples over 1 h were tested at 25 °C, a scanning strain of 1%, and a frequency of 0.5 Hz.

### 2.4. Texture Test

Gelatinized samples were cooled at room temperature, sealed and stored in a refrigerator (4 °C) for 24 h. Texture analysis of the samples was performed using the texture analyzer (CT3, Brookfield, Middleboro, MA, USA) with a P/0.5 probe, at 1.0 mm/s pretest rate, 1.0 mm/s test rate, 1.0 mm/s return rate, 50% compression, and with a 5 g trigger force. Each group of samples was tested ten times.

### 2.5. Microstructure Observation

The gel microstructure was observed by scanning electron microscopy (SEM; JSM-6510LV, JEOL, Tokyo, Japan). Firstly, gelatinized samples were uniformly poured into the culture dish and sealed with preservative films. The samples were then frozen at −18 °C for 24 h and then dried in a vacuum freeze drier (FD-1A-50, Boyikang Laboratory Instruments, Beijing, China) for 36 h, in a 0.255 mbar vacuum, and a panel temperature of 15 °C. Dried samples were fixed on the sample table by double-sided adhesive tape, and electrical conductivity was obtained through ion sputtering metal spraying. Then, the samples were observed via SEM at 20 kV and ×200 magnification.

### 2.6. Data Processing

All experiments were performed in triplicate and data were expressed as the mean ± standard deviation. Related graphs were drawn using Origin 8.6 (SPSS Inc., Chicago, IL, USA), and analysis of variance (ANOVA) was conducted using SPSS 11.5 (MicroCal Software Inc., Northampton, MD, USA). Differences among different means were compared using Duncan’s method (*p* < 0.05).

## 3. Results and Analysis

### 3.1. Pasting Properties

**Figure 1:**
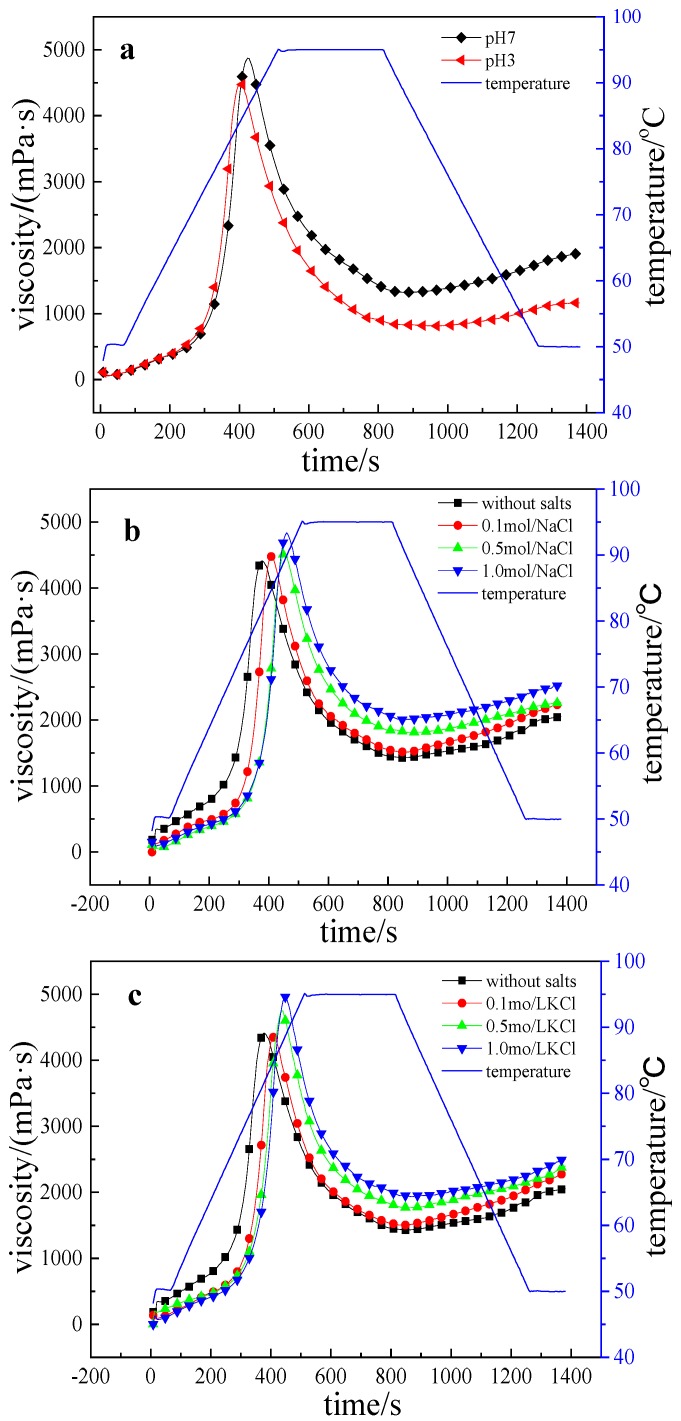

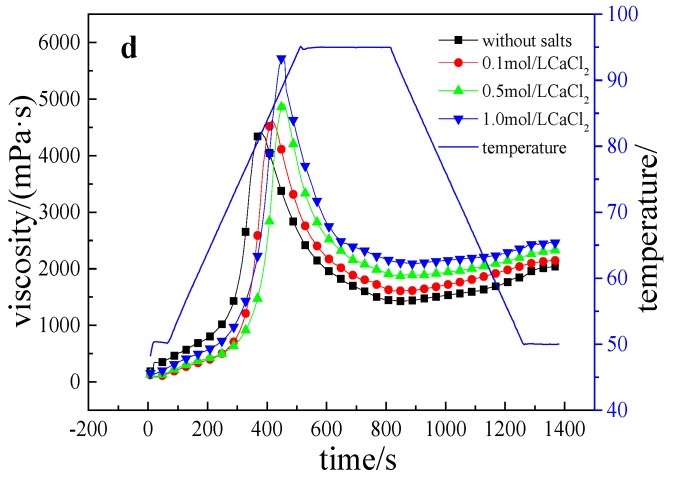
Pasting curves of LRS/KGM mixtures in acid or salt solutions. (**a**) acid (citric acid buffer); (**b**) NaCl; (**c**) KCl; (**d**) CaCl_2_.

Pasting is a characteristic that reflects the starch behavior when heated with excess water, and viscosity change during the process could give information about starch suitability for industrial application [21]. The pasting curves and eigenvalues of the LRS/KGM mixture at pH 7 and 3 are shown in Figure 1a and Table 1, respectively. The results show that the LRS/KGM mixture had a lower peak viscosity, final viscosity, setback viscosity, pasting time, peak time, and a higher breakdown viscosity in acidic conditions than in a neutral environment. Given that acid hydrolysis destroys glycosidic bonds in molecules [22], the complete pasting of starch molecules in acidic conditions requires less energy and therefore LRS is gelatinized faster and at lower temperatures. The hydrolysis of the LRS/KGM mixture in acidic environments is irreversible and influences the rearrangement of starch molecules upon temperature reduction, accompanied by a decrease in the retrogradation rate and value. Following the decomposition of long-chain to short-chain starch molecules [19], the shear stress capacity of the mixture decreases, with a subsequent increase in breakdown viscosity during the pasting and stirring processes. Under a constant stirring rate, there is a decrease in shear resistance, with minimal resistance being exerted against the rotation of the stirring blades, as manifested by a reduction in peak and final viscosities. Indeed, this finding conforms to the results of tapioca starch/xanthan gum mixtures in acid solution [19].

The pasting curves of the LRS/KGM mixture in NaCl, KCl and CaCl_2_ solutions are shown in Figure 1b–d, respectively, whereas the pasting eigenvalues are listed in Table 1. The three salt types exert similar effects on the pasting of the LRS/KGM mixture despite the different degrees of impact. Salt addition was positively correlated with an increased peak viscosity, final viscosity, breakdown viscosity, pasting temperature, and peak time of the LRS/KGM mixture, but decreased the setback viscosity. Given that all three salt types are strong electrolytes, the metal cations (e.g., Na^+^, K^+^ and Ca^2+^) and the anion (Cl^−^) in the solutions influence the interaction between starch and water molecules [18], thus destroying the starch–water and starch–starch hydrogen bonds and consequently inhibiting the pasting process. Furthermore, the effects of NaCl and KCl on the pasting of the mixture were similar. Conversely, CaCl_2_ had a more significant influence on pasting due to the crosslinking of Ca^2+^ within the gel and its stronger hydrability [23,24]. Thus, Ca^2+^ is able to interact with starch and water more strongly, hindering the expansion of starch particles and exerting a greater inhibition on the system during pasting.

Furthermore, the water activity of the LRS/KGM mixture was weakened following the addition of salt, likely due to a decrease in the penetration of water molecules within starch particles during the heating process, thus hindering pasting, and leading to an increase in the pasting temperature of starch with a delay in peak time. Indeed, the inhibition of water molecule penetration is known to be greater with the increasing ionic concentration [17]. Therefore, the pasting temperature and peak time improves regularly with the increase in salinity. With the additional increase in salinity, the breakdown and final viscosities of LRS were observed to increase gradually, due to salt–starch interactions reducing the mobility of the starch molecular chain segment [15], thereby increasing the peak and final viscosities. Moreover, LRS molecules adsorb ions from the salt solution, leading to partial particle expansion and a subsequent increase in particle volume. Thus, starch particles are easily destroyed by shear stress, leading to an increase in breakdown viscosity. In addition, salt ions disrupt the hydrogen bonding between starch molecules and between starch and KGM, hindering the rearrangement of starch molecules in static conditions and at low temperature [18], and thus reducing the setback viscosity.

### 3.2. Static Shear Rheology

**Figure 2:**
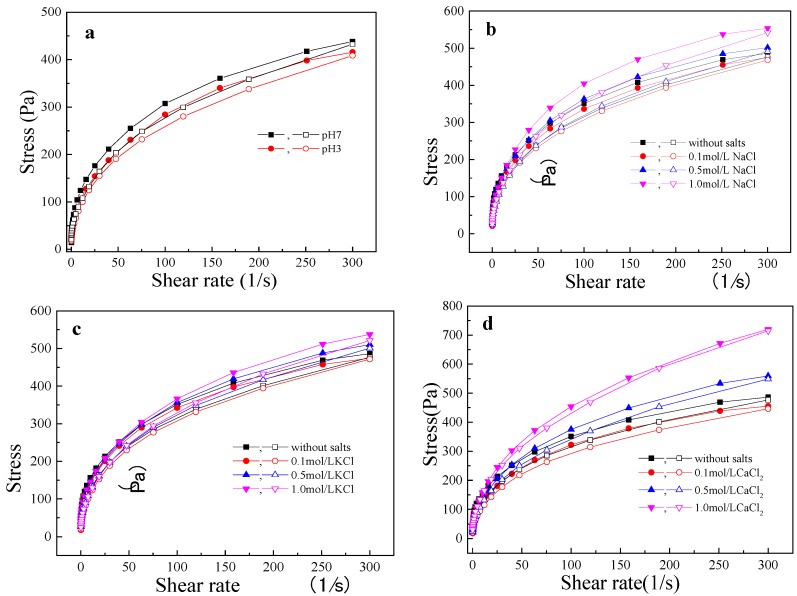
Flow curves of LRS/KGM mixtures in acid or salt solutions. (**a**) acid (citric acid buffer); (**b**) NaCl; (**c**) KCl; (**d**) CaCl_2_. Closed symbols denote the uplink line (the curve of the shear force applied to the sample when the shear rate is varied from 0 s^−1^ to 300 s^−1^) and opened symbols denote the downlink line (the curve of the shear force applied to the sample when the shear rate varies from 300 s^−1^ to 0 s^−1^).

The shear stress versus shear rate curves for the LRS/KGM mixture at pH 7 and 3 are shown in Figure 2a. At both pH values, shear stress was positively correlated with shear rate. The shear stress of the LRS/KGM mixture at pH 3 was lower than that at pH 7, indicating that acidic conditions weaken the shear resistance of the system. Regression fitting of curve data points was conducted based on the power law equation (Table 2). According to the fitted data, the multiple correlation coefficient (*R*^2^) was more than 0.99, indicating that the power law equation is applicable for the rheological curves and that the mixture exhibited a significant difference in rheological properties between pH 3 and pH 7 (*p* < 0.05). Furthermore, a *n* < 1 fluid index was calculated and thus the system was classified as a pseudoplastic fluid (Table 2). The smaller the value of *n*, the stronger the pseudoplasticity or shear thinning [25]. At pH 3, *K* was calculated to be smaller than at pH 7, but *n* was higher, indicating that the LRS/KGM mixture underwent a weak thickening effect and poor pseudoplasticity in an acid environment. Following starch molecule hydrolyzation into micromolecular segments at low pH, the number of macromolecules in the starch chain, as well as the interaction between LRS and KGM molecular chains, decreased, resulting in a reduction of shear stress and of the consistency coefficient. At high-speed shear stress, the macromolecular structure of the main chain in the LRS/KGM mixture underwent an oriented arrangement, reducing flow resistance and causing shear thinning [26]. However, upon macromolecule hydrolyzation, the original oriented arrangement structure was disrupted, weakening the shear thinning effect.

The shear stress versus shear rate curves of the LRS/KGM mixture in NaCl, KCl, and CaCl_2_ solutions are shown in Figure 2b–d, respectively. The three salt solutions exerted similar effects on the LRS/KGM mixture at the various concentrations. Compared with the LRS/KGM mixture without salt, the mixture with 0.1 mol/L of salt (for all three types) had a lower shear stress, whereas the mixtures with 0.5 and 1 mol/L of salt showed a higher shear stress. Thus, shear stress was also positively related to salinity, with an improved shear resistance observed at higher salt concentrations. The power law fitting results of the curves are listed in Table 2. The increase in salinity respectively increased the *K* and *n* values of the LRS/KGM mixture, indicating that salt addition improves the thickening effect of the system but weakens pseudoplasticity. Further, the internal electrostatic interaction forces of the system were significantly increased due to the presence of salt ions [24]. It is likely that the types and strength of the internal electrostatic interactions within the system were enhanced with the increase in salinity, thus increasing the value of *K*. Alternatively, saline ions may have facilitated interactions between LRS and KGM molecules, enhancing the forces acting against flow [27] and therefore weakening shear thinning. Compared with NaCl and KCl, CaCl_2_ led to a greater increase in shear resistance and thickening effect within the system. Given that divalent cations are known to exert a greater influence on starch/hydrophilic colloid interactions than monovalent cations [28].

### 3.3. Dynamic Viscoelastic Rheology

**Figure 3:**
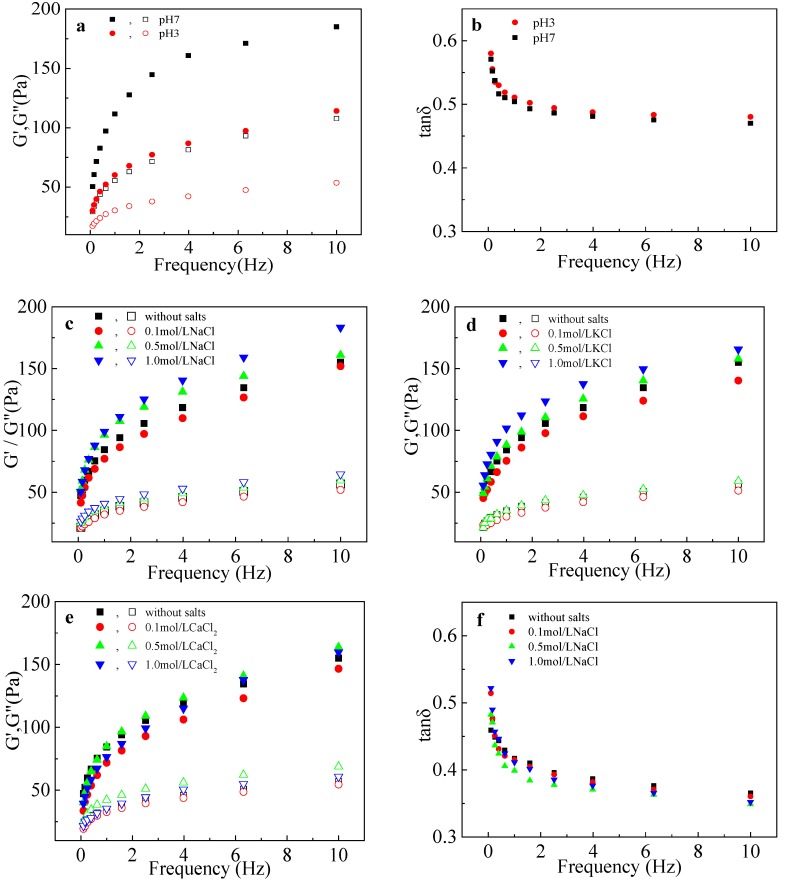

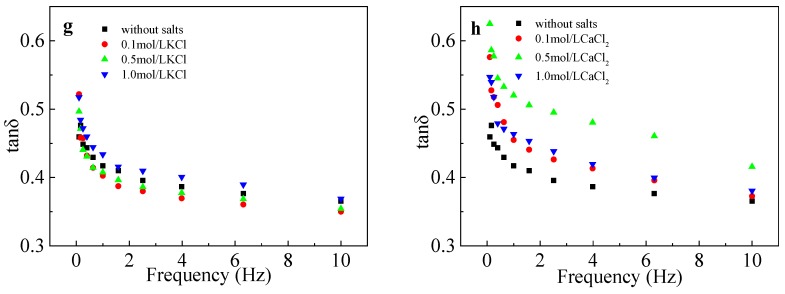
Curves of changes in dynamic modulus and tan δ of LRS/KGM mixtures with frequency in acid or salt solutions. Closed symbols denote the storage modulus (*G*′) and opened symbols denote the loss modulus (*G*″). (**a**,**b**) acid (citric acid buffer); (**c**,**f**) NaCl; (**d**,**g**) KCl; (**e**,**h**) CaCl_2_.

The variations in *G*′, *G*″, and tan δ of the LRS/KGM mixture with frequency at pH 3 and 7 are shown in Figure 3a,b, respectively. *G*′ and *G*″ can be used to express the elasticity and viscosity of a material upon deformation. In an acidic environment, the elasticity and viscosity of the LRS/KGM mixture were smaller than those in a neutral environment. Tan δ is equal to *G*″/*G*′; thus, if tan δ is close to 1, the given mixture will have a strong viscosity [25]. The LRS/KGM mixture showed a higher tan δ in an acidic environment compared to a neutral environment, indicating that an acidic environment is able to enhance the viscosity of the system rather than the elasticity. Within the discussed system, starch was hydrolyzed into smaller molecular segments under acidic conditions, thus reducing the interactions between the internal molecular chain and destroying the network structure [20], thereby reducing the elasticity.

The variation curves of *G*′ and *G*″ of the LRS/KGM mixture with frequency in NaCl, KCl, and CaCl_2_ solutions are shown in Figure 3c–e, respectively. *G*′ and *G*″ showed a high frequency dependence in the frequency range of 0–10 Hz. With the increase in frequency, *G*′ values for all systems were higher than the corresponding *G*″ values, indicating that the viscoelasticity was dominated by elasticity. Compared with the unsalted LRS/KGM mixture, the LRS/KGM mixture in a 0.1 mol/L salt solution had lower *G*′ and *G*″ values, indicating a reduced viscoelasticity. Under this salt concentration, the inhibitory effect of the metal ions on hydrogen bond formation between starch molecules remained stronger than the electrostatic interactions induced by the metal ions [29]. With a salinity increase to 0.5 and 1 mol/L, the metal ions were able to penetrate the starch and KGM molecular chains and change the molecular conformation, increasing the entanglement points and strengthening the electrostatic interactions, thus increasing the *G*′ and *G*″ values [30]. Therefore, the most significant increases in *G*′, *G*″, and the highest viscoelasticity were achieved using the 1 mol/L NaCl and 1 mol/L KCl solutions. Nevertheless, with the 0.5 mol/L CaCl_2_ solution, the *G*′ and *G*″ values were the highest, likely due to the special divalent charge of Ca^2+^.

The variations in tan δ with frequency in the NaCl, KCl and CaCl_2_ solutions are shown in Figure 3f–h, respectively. Tan δ is closely related to frequency. For all systems, tan δ decreased continuously with the increase in frequency, indicating the formation of elastic solids under high-frequency oscillations. The tan δ of LRS/KGM mixtures at the various NaCl concentrations was similar but lower than that of the unsalted system. The minimum tan δ value was achieved at a 0.5 mol/L NaCl concentration, indicating that the 0.5 mol/L NaCl solution can increase the proportion of elasticity in the LRS/KGM mixture, likely due to the induction of KGM molecular aggregates by NaCl, in agreement with a previous study concerning the effects of NaCl on the rheology of a sweet potato–xanthan gum mixture [15]. Moreover, tan δ increased continuously with the increase in KCl, exceeding that of the unsalted system at 1 mol/L. Given that KCl was able to increase the viscoelasticity of the system at this concentration, the viscosity growth amplitude was higher than the elasticity growth amplitude. In the CaCl_2_ systems, tan δ was larger than that of the unsalted system, indicating that CaCl_2_ significantly enhanced the viscosity of the LRS/KGM mixture, and reaching a peak at 0.5 mol/L, also likely due to the special divalent charge.

### 3.4. Dynamic Time Scanning

**Figure 4:**
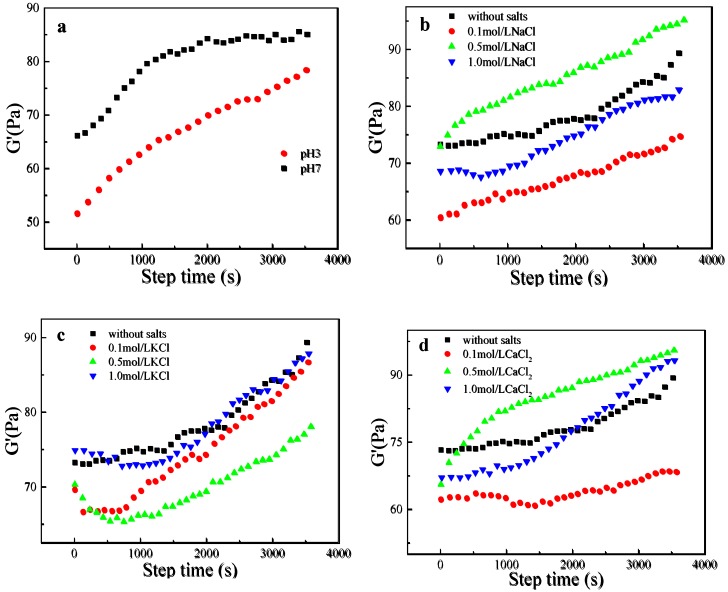
Curves of change in the storage modulus of LRS/KGM mixtures with time in acid or salt solutions. (**a**) acid (citric acid buffer); (**b**) NaCl; (**c**) KCl; (**d**) CaCl_2_.

The *G*′ value versus time curves of the LRS/KGM mixture after 1 h heat gelatinization at pH 3 and 7 are shown in Figure 4a. Changes in *G*′ values during aging retrogradation reflect the structure formation rate of a system [31]. The increase in *G*′ values was attributed to the fast aggregation of amylose in the early retrogradation period and the slow aggregation of amylopectin in the late retrogradation period [31]. At pH 3, the value of *G*′ increased continuously with time, while at pH 7, *G*′ increased rapidly during the first 1500 s, with a gradual deceleration and subsequently reaching a stable value. Nevertheless, the value of *G*′ at pH 3 remained lower than that at pH 7 throughout the test, indicating that the LRS/KGM mixture presents a weaker short-term retrogradation at pH 3 than at pH 7. Acid hydrolysis is also the main reason leading to a lower *G*′ value in this system [32].

The *G*′ value versus time curves of the LRS/KGM mixture after 1 h heat gelatinization in solutions of varying NaCl, KCl and CaCl_2_ concentrations are shown in Figure 4b–d, respectively. The *G*′ value continued to increase over 1 h, and the various salt types had a different degree of impact on the *G*′ values of the LRS/KGM mixture. In LRS/KGM mixtures with NaCl, the *G*′ value increased with time. However, no significant relationship between *G*′ values and NaCl concentration was observed. The *G*′ value of the LRS/KGM mixture in 0.5 mol/L of NaCl was higher than those at 0.1 and 1 mol/L, as well as than the *G*′ value of the unsalted system. The *G*′ values for 0.1 and 1 mol/L NaCl were both lower than that of the unsalted system. Thus, the LRS/KGM mixture in 0.5 mol/L of NaCl achieved the highest formation rate of the internal 3D structure, likely due to exhibiting the strongest molecular interactions and rearrangement. A previous study also analyzed the influence of NaCl concentrations on the *G*′ value of sago starch, determining that the highest *G*′ value is achieved at a 0.5 mol/L NaCl concentration [33]. The *G*′ value of the LRS/KGM mixture in KCl solutions led to a V-shaped variation with time. In the 0.1 and 1.0 mol/L KCl solutions, the *G*′ values were close to that of the unsalted system; however, in the 0.1 mol/L KCl solution, the increase rate of *G*′ and the formation rate of the 3D network were both higher. The *G*′ value in the 0.1 mol/L CaCl_2_ solution was relatively low, whereas a rapid increase was observed in the 0.5 and 1 mol/L solutions, exceeding that of the unsalted system; indeed, the maximum *G*′ value was achieved in the 0.5 mol/L CaCl_2_ solution. Thus, the varying effects of salt type on the LRS/KGM mixture are closely related to the charge and concentration of the internal cations. For a given salt, complexation between the metal ions and hydroxyls inhibits the formation of amylose aggregates at a given solution concentration according to the salt used [33], thus increasing the *G*′ value.

### 3.5. Gel Texture Test

The gel texture parameters of the LRS/KGM mixture in acidic and saline environments are presented in Table 3. The gel hardness, cohesiveness, elasticity and adhesiveness in an acidic environment were lower than those in a neutral environment. Texture is also positively correlated with hydrogen bond formation and the ordered arrangement of starch molecules [34]. Acid hydrolysis breaks the molecular chain of starch, weakening the intermolecular interactions and disrupting molecular arrangements, and therefore reducing the texture parameters, especially with regards to hardness.

Notably, the hardness of the LRS/KGM mixture in salt solutions was lower than that in the unsalted system, albeit with a significantly higher cohesiveness (*p* < 0.05). The variation of elasticity and adhesiveness was associated with salt type and concentration. A strong amylose molecular interaction (mostly hydrogen bonds) results in high starch gel hardness [34,35]. Metal ions disrupt molecular interactions and react with hydroxyl groups in starch to produce complexation, which also hinders amylose aggregation to some extent [33,35]. Thus, the hardness of the LRS/KGM system decreases after salt addition. However, among the range of decreased hardness, probably due to “salting-out” or “salting-in” effect, there was a critical concentration, where the hardness was higher than for other concentrations of the same salt solution [24,28]. In the LRS/KGM mixture, the critical salt concentrations were 0.5 mol/L NaCl, 0.1 mol/L KCl and 0.5 mol/L CaCl_2_, respectively. This finding conforms to the dynamic time scanning results stated above. Cohesiveness, which reflects the degree of difficulty in breaking down the gel’s internal structure [28], increased following the addition of salt, indicating an increase in the internal bond strength of the mixture. Moreover, the change in elasticity and adhesiveness were positively related to the concentrations of NaCl, KCl and CaCl_2_. At the same given concentration, the LRS/KGM mixture in CaCl_2_ achieved the highest elasticity and adhesiveness, likely due to the strong interaction of high-valence calcium ions [23,24] as well as the crosslinking of coordinate bonds between polyvalent metal ions and KGM molecules [36]. Similar conclusions were obtained when assessing the influence of NaCl and CaCl_2_ on the texture of potato starch gel [24].

### 3.6. Microstructure Observation

**Figure 5:**
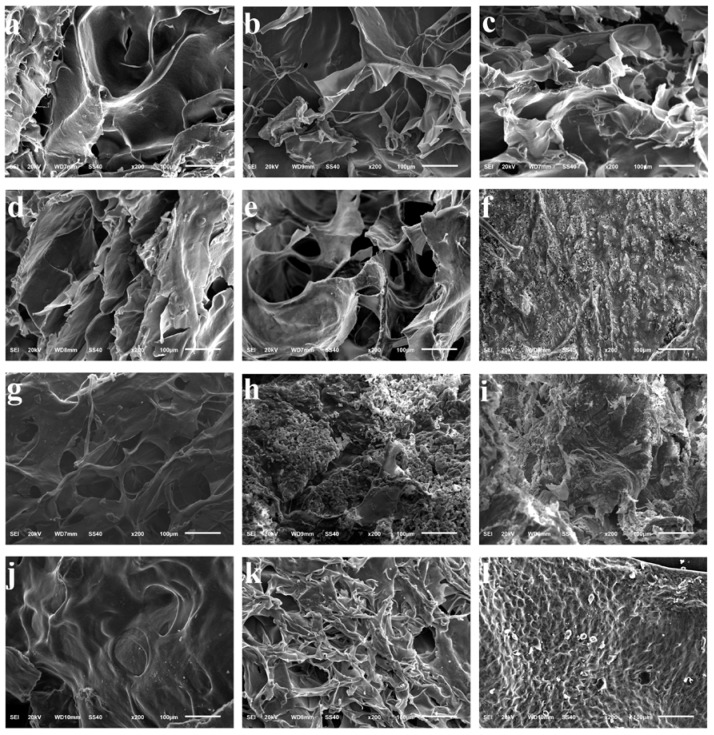
Microstructure of LRS/KGM mixtures in acid or salt solutions at pH 7 (**a**), pH 3 (**b**), without salts (**c**), 0.1 mol/L NaCl (**d**), 0.5 mol/L NaCl (**e**), 1.0 mol/L NaCl (**f**), 0.1 mol/L KCl (**g**), 0.5 mol/L KCl (**h**), 1.0 mol/L KCl (**i**), 0.1 mol/L CaCl_2_ (**j**), 0.5 mol/L CaCl_2_ (**k**) and 1.0 mol/L CaCl_2_ (**l**).

The SEM images of the LRS/KGM mixture in acidic (pH 3) and neutral (pH 7) environments are shown in Figure 5a,b, respectively. The LRS/KGM mixture had a continuous gel network with some small pores in the neutral environment, but developing a ruptured network with larger pores in the acidic environment. The starch molecular chains were hydrolyzed at pH 3, damaging the original network, and subsequently leading to rupture and large pore formation. This finding is consistent with those of a tapioca starch–xanthan gum mixture, albeit with different reaction conditions [20].

SEM images of the LRS/KGM mixture gel structures following the addition of salts are shown in Figure 5c–l. It revealed that the gel structures were damaged by the addition of salts. The unsalted LRS/KGM mixture exhibited a honeycomb-like structure with some small pores (Figure 5c). When the NaCl concentration was increased from 0.1 mol/L to 1 mol/L (Figure 5d–f), the structure underwent network collapse, network reformation and network disappearance. In the 0.1 mol/L NaCl solution, the Na^+^ ions led to a disruption of hydrogen bonding and a significant enhancement of electrostatic repulsion, inducing structure destruction. In the 0.5 mol/L NaCl solution, the above interactions were redistributed, as stated before, resulting in a reformed gel network and honeycomb-like structure, with a pore size enlargement, along with a slight increase in hardness (Table 3). In the 1 mol/L NaCl solution, the damage by NaCl reached saturation and excessive electrostatic interactions forced Na^+^ ion adsorption and subsequent aperture and pore disappearance, resulting in a destroyed gel network, with a consequential decrease in hardness (Table 3). Similar results were found in a similar system of a mesona blumes gum/rice starch mixed gel following the addition of NaCl [28].

When the KCl concentration increased from 0.1 mol/L to 1 mol/L (Figure 5g–i), the pore number and size gradually decreased until they finally disappeared, with the gel network collapse. Different from the honeycomb-like structure developed in the 0.5 mol/L NaCl system, the structure in the KCl system was continuously destroyed at 0.5 mol/L; and at 1 mol/L of KCl, a greater number of K^+^ ions were adsorbed on the surface, developing a more collapsed network than with 1 mol/L of NaCl. Consequentially, gel hardness decreased even further than 1 mol/L NaCl (Table 3).

The LRS/KGM mixtures in 0.1, 0.5 and 1 mol/L CaCl_2_ are shown in Figure 5j–l, respectively. With the increase in CaCl_2_ concentration, a process of network collapse, reformation and disappearance was also underwent in the gel structure, similar to the observations in the NaCl solution. In the 0.5 mol/L of CaCl_2_ solution, the system developed a more compact network structure, in agreement with the hardness results (Table 3). In contrast to the NaCl and KCl systems, the structures of the CaCl_2_ system had smaller pores and a tighter gel network, illustrating that the Ca^2+^ interactions were stronger than those by Na^+^ and K^+^.

## 4. Conclusions

The pasting eigenvalues (except for breakdown viscosity) and rheological parameters (except for fluid index) of the LRS/KGM mixtures decreased significantly in an acidic environment, accompanied by weakened retrogradation, thickening, pseudoplasticity and viscoelasticity. Moreover, the gel texture and microstructure deteriorated. In a salt environment, the peak, final and breakdown viscosity, pasting temperature, and peak time of the LRS/KGM mixtures exhibited a regular increase with the increase in NaCl, KCl and CaCl_2_ concentrations, except for the setback viscosity. Furthermore, salinity was able to enhance the thickening of the mixtures and decrease pseudoplasticity and retrogradation. In addition, salt type and concentration were able to influence the viscoelasticity, texture and microstructures of the LRS/KGM mixtures to different extents. The results of this study could provide a reference for the development and application of other starch products. For example, the thickening effect of LRS/KGM mixtures can be enhanced by the addition of 1 mol/L salt solutions, whereas hard gels can be formed by the addition of 0.5 mol/L NaCl and CaCl_2_ solutions.

## Figures and Tables

**Table 1:** Pasting parameters of the LRS/KGM mixed system in acid or salt solutions.

Sample		Peak viscosity (mPa·s)	Final viscosity (mPa·s)	Breakdown viscosity (mPa·s)	Setback viscosity (mPa·s)	Peak time (min)	Pasting temperature (°C)
Acid (citric acid buffer)	pH 3	4493.5 ± 10.4 ^b^	1163.5 ± 5.3 ^b^	3680.5 ± 5.3 ^a^	350.5 ± 3.1 ^b^	6.73 ± 0.24 ^a^	67.72 ± 1.23 ^a^
	pH 7	4880.0 ± 12.3 ^a^	1914.5 ± 7.2 ^a^	3559.5 ± 8.7 ^a^	594.0 ± 2.0 ^a^	7.10 ± 0.17 ^a^	69.47 ± 0.97 ^a^
Salt	Without salts	4410.5 ± 14.1 ^e^	2053.4 ± 6.0 ^e^	2942.1 ± 10.3 ^b^	998.6 ± 10.2 ^a^	6.27 ± 0.12 ^c^	51.95 ± 0.74 ^c^
	0.1 mol/L NaCl	4476.4 ± 19.3 ^e^	2240.5 ± 4.4 ^c^	2996.4 ± 9.5 ^b^	760.5 ± 8.4 ^b^	6.81 ± 0.07 ^b^	65.75 ± 1.04 ^b^
	0.5 mol/L NaCl	4526.5 ± 7.8 ^d^	2263.6 ± 7.5 ^c^	3017.3 ± 6.6 ^b^	455.7 ± 5.4 ^e^	7.53 ± 0.25 ^a^	72.75 ± 2.33 ^a^
	1.0 mol/L NaCl	4835.5 ± 20.4 ^b^^c^	2513.9 ± 8.6 ^a^	3025.6 ± 9.4 ^b^	510.5 ± 7.3 ^d^^e^	7.67 ± 0.09 ^a^	73.75 ± 2.18 ^a^
	0.1 mol/L KCl	4417.8 ± 11.2 ^e^	2294.6 ± 8.3 ^b^^c^	2947.5 ± 8.2 ^b^	793.9 ± 3.6 ^b^	6.83 ± 0.14 ^b^	66.05 ± 1.34 ^b^
	0.5 mol/L KCl	4747.6 ± 7.4 ^c^	2393.9 ± 4.9 ^ab^	2981.2 ± 10.2 ^b^	627.4 ± 4.5 ^c^	7.27 ± 0.22 ^a^	70.65 ± 1.45 ^a^
	1.0 mol/L KCl	4961.5 ± 14.2 ^b^	2494.0 ± 2.8 ^a^	3027.5 ± 14.3 ^b^	559.6 ± 6.5 ^d^	7.47 ± 0.04 ^a^	71.43 ± 2.40 ^a^
	0.1 mol/L Ca_2_Cl	4615.7 ± 12.1 ^c^^d^	2152.5 ± 5.0 ^d^	3009.1 ± 11.2 ^b^	544.6 ± 3.4 ^d^	6.93 ± 0.09 ^b^	67.45 ± 2.21 ^b^
	0.5 mol/L Ca_2_Cl	4919.0 ± 15.8 ^b^	2331.7 ± 9.4 ^b^	3043.4 ± 13.3 ^b^	456.3 ± 9.5 ^e^	7.60 ± 0.32 ^a^	71.91 ± 2.72 ^a^
	1.0 mol/L Ca_2_Cl	5721.5 ± 10.1 ^a^	2461.4 ± 10.7 ^a^	3773.2 ± 10.4 ^a^	513.5 ± 5.8 ^d^^e^	7.47 ± 0.21 ^a^	72.13 ± 1.29 ^a^

Note: Letters on the average value in the same row indicates significant difference (*p* < 0.05).

**Table 2:** Power law parameters of the LRS/KGM mixed system in acid or salt solutions.

Sample		Uplink line			Downlink line		
		*K* (Pa·s^n^)	*n*	*R^2^*	*K* (Pa·s^n^)	*n*	*R^2^*
Acid (citric acid buffer)	pH 3	44.564 ± 1.224 ^b^	0.395 ± 0.005 ^a^	0.997 ± 0.001 ^a^	35.919 ± 0.652 ^b^	0.427 ± 0.003 ^a^	0.995 ± 0.002 ^a^
	pH 7	53.389 ± 1.103 ^a^	0.372 ± 0.004 ^b^	0.991 ± 0.002 ^a^	40.578 ± 0.839 ^a^	0.415 ± 0.004 ^b^	0.992 ± 0.001 ^a^
Salt	Without salts	64.647 ± 1.618 ^c^	0.323 ± 0.004 ^d^	0.998 ± 0.001 ^a^	42.367 ± 1.025 ^d^	0.379 ± 0.005 ^c^	0.998 ± 0.001 ^a^
	0.1 mol/L NaCl	65.177 ± 1.182 ^c^	0.351 ± 0.003 ^c^	0.998 ± 0.002 ^a^	45.178 ± 0.960 ^c^	0.380 ± 0.003 ^c^	0.992 ± 0.001 ^a^
	0.5 mol/L NaCl	68.271 ± 1.848 ^b^	0.349 ± 0.005 ^c^	0.997 ± 0.001 ^a^	48.843 ± 0.992 ^b^	0.405 ± 0.004 ^b^	0.999 ± 0.002 ^a^
	1.0 mol/L NaCl	69.256 ± 2.606 ^b^	0.374 ± 0.007 ^b^	0.996 ± 0.003 ^a^	52.241 ± 1.208 ^a^	0.422 ± 0.004 ^a^	0.998 ± 0.001 ^a^
	0.1 mol/L KCl	65.647 ± 1.652 ^c^	0.345 ± 0.005 ^c^	0.997 ± 0.002 ^a^	45.960 ± 0.830 ^c^	0.400 ± 0.003 ^b^	0.999 ± 0.003 ^a^
	0.5 mol/L KCl	66.668 ± 1.466 ^c^	0.359 ± 0.004 ^c^	0.998 ± 0.001 ^a^	47.962 ± 0.917 ^b^	0.407 ± 0.003 ^b^	0.990 ± 0.003 ^a^
	1.0 mol/L KCl	67.302 ± 1.598 ^b^^c^	0.371 ± 0.004 ^b^	0.993 ± 0.002^a^	49.326 ± 0.925 ^b^	0.421 ± 0.003 ^a^	0.998 ± 0.002 ^a^
	0.1 mol/L Ca_2_Cl	66.381 ± 1.702 ^c^	0.381 ± 0.006 ^b^	0.997 ± 0.001 ^a^	47.844 ± 0.961 ^b^	0.408 ± 0.004 ^b^	0.998 ± 0.003 ^a^
	0.5 mol/L Ca_2_Cl	72.193 ± 1.540 ^b^	0.410 ± 0.005 ^a^	0.998 ± 0.002 ^a^	53.416 ± 0.885 ^a^	0.429 ± 0.003 ^a^	0.991 ± 0.003 ^a^
	1.0 mol/L Ca_2_Cl	77.592 ± 0.978 ^a^	0.438 ± 0.003 ^a^	0.994 ± 0.001 ^a^	56.162 ± 1.281 ^a^	0.445 ± 0.004 ^a^	0.993 ± 0.001 ^a^

Note: Letters on the average value in the same row indicates significant difference (*p* < 0.05).

**Table 3:** Texture Parameters of the LRS/KGM mixed system in acid or salt solutions.

Sample		Hardness (g)	Cohesiveness	Elasticity (mm)	Adhesiveness (mJ)
Acid (citric acid buffer)	pH 3	80.11 ± 2.46 ^b^	0.42 ± 0.02 ^b^	12.70 ± 0.18 ^b^	0.81 ± 0.01 ^b^
	pH 7	100.02 ± 12.29 ^a^	0.47 ± 0.02 ^a^	13.27 ± 0.15 ^a^	0.91 ± 0.04 ^a^
Salt	Without salts	105.34 ± 10.21 ^a^	0.45 ± 0.04 ^e^	12.97 ± 0.28 ^b^	0.71 ± 0.15 ^b^
	0.1 mol/L NaCl	87.69 ± 3.57 ^b^	0.57 ± 0.04 ^d^	12.66 ± 0.58 ^b^	0.65 ± 0.15 ^b^
	0.5 mol/L NaCl	92.32 ± 6.33 ^b^	0.54 ± 0.05 ^d^	12.96 ± 0.74 ^b^	0.75 ± 0.01 ^b^
	1.0 mol/L NaCl	85.20 ± 3.22 ^b^	0.63 ± 0.02 ^c^	13.42 ± 0.17 ^a^^b^	0.84 ± 0.12 ^a^^b^
	0.1 mol/L KCl	67.89 ± 5.37 ^c^	0.61 ± 0.01 ^c^	12.73 ± 0.48 ^b^	0.70 ± 0.05 ^b^
	0.5 mol/L KCl	61.42 ± 3.91 ^c^	0.66 ± 0.07 ^bc^	12.84 ± 0.73 ^b^	0.73 ± 0.21 ^b^
	1.0 mol/L KCl	36.73 ± 2.47 ^d^	0.87 ± 0.11 ^a^	14.76 ± 0.84 ^a^	0.85 ± 0.22 ^a^^b^
	0.1 mol/L Ca_2_Cl	67.48 ± 3.99 ^c^	0.61 ± 0.06 ^c^	12.90 ± 0.13 ^b^	0.73 ± 0.25 ^b^
	0.5 mol/L Ca_2_Cl	72.68 ± 4.53 ^c^	0.71 ± 0.03 ^b^	13.48 ± 0.54 ^ab^	1.15 ± 0.15 ^a^
	1.0 mol/L Ca_2_Cl	49.39 ± 3.58 ^d^	0.75 ± 0.02 ^b^	14.85 ± 0.45 ^a^	1.26 ± 0.13 ^a^

Note: Letters on the average value in the same row indicates significant difference (*p* < 0.05).
